# The Use of Virtual Microscopy and a Wiki in Pathology Education: Tracking Student Use, Involvement, and Response

**DOI:** 10.1155/2014/274134

**Published:** 2014-11-23

**Authors:** Zev Leifer

**Affiliations:** New York College of Podiatric Medicine, New York City, NY 10035, USA

## Background

The Pathology Laboratory course at the New York College of Podiatric Medicine involves the use of Virtual Microscopy. As with traditional microscopes, the students can scan the whole slide, section by section, and zoom in or out for further detailed study. Using the advantages of Digital Pathology, the students can, in addition, access the slide collections from other medical schools and put up normal histology (control) slides side-by-side with the pathology. They cut and paste and preserve the ROI that they find. They edit and annotate (circle, underline, label, add arrows, etc.). The creation of a wiki, its application, and the student response to it are yet another level of preprofessional training to the above.

## Method

A wiki was created (http://pathlab2014.wikifoundry.com/) for the Class of 2014. The students saved, edited, and uploaded their slides. In the wiki format, other students could comment, further edit, and even delete the slides.

## Results

216 images were uploaded. These were available in one full presentation. They were also grouped into sixteen albums, grouped by the topics in Systemic Pathology. They were available to all. The student access was followed by Google Analytics. This provided a detailed analysis of student use. At the end of the course, a questionnaire was distributed, assessing their impression of the wiki format and soliciting strengths and weaknesses. Over one hundred sets of comments were obtained. Most comments were favorable, describing particular features that they liked, such as ease of use, sharing of notes, aids in exam preparation, and permission for students to work together. Negative comments were mostly directed to technical issues of accessing and using the site.

## Conclusions

The use of a wiki as described has a number of important advantages in Pathology Education. It trains the students in the more sophisticated skills that they will use as professional pathologists or as clinicians: (1) Telepathology—it enables them to gain skill in putting up slides on the internet, sharing and discussing the observations. This parallels the process of Tumor Boards and consultation for group consensus. (2) Archiving and Retrieval—it models the challenge faced by hospitals, diagnostic labs, and physicians in maintaining a collection of slides in a form that is easily accessible, always available, and universally sharable. With the album feature, slides can be multiply stored, by patient name, by pathology, by tissue type, and so on. (3) Image Analysis—while not generally used in an undergraduate lab, familiarity with a wiki format allows them to jump easily to capturing and storing images found in the literature or provided by colleagues or the diagnostic lab pathologist's report that have been stained for tumor markers or other diagnostic staining techniques.

Experience with the use of a wiki in Pathology Education has been quite satisfactory from both the faculty and the student's point of view. It has a number of training advantages in the ever-expanding world of Digital Pathology.

